# Portable wide-field femtoliter-chamber imaging system for point-of-care digital bioanalysis

**DOI:** 10.1016/j.isci.2024.110868

**Published:** 2024-09-01

**Authors:** Tatsuya Iida, Jun Ando, Mami Yoshimura, Asami Makino, Masahiro Nakano, Yasushi Kogo, Hajime Shinoda, Masashi Toyoda, Takeshi Noda, Rikiya Watanabe

**Affiliations:** 1 Molecular Physiology Laboratory, Cluster for Pioneering Research, RIKEN, Saitama, Japan; 2 Institute for Life and Medical Sciences, Kyoto University, Kyoto, Japan; 3 Tokyo Metropolitan Institute for Geriatrics and Gerontology, Tokyo, Japan

**Keywords:** Applied sciences, Bioelectronics, Biotechnology

## Abstract

Recently, digital bioanalysis using femtoliter (fL)-chamber arrays has significantly improved the sensitivity, accuracy, and throughput of conventional nucleic acid and antigen tests, with great potential for the diagnosis of infectious diseases and underlying disorders. However, the large size of conventional platforms with costly assay consumables for digital bioanalysis complicates its use in point-of-care testing (POCT). To solve these problems, in this study, we developed a wide-field fL-chamber imaging system (COWFISH2), a portable wide-field femtoliter-chamber imaging system (footprint: 14 × 22 cm), by redesigning various electronic controls and optical systems of COWFISH, accompanied by the development of low-cost and durable consumables for digital bioanalysis. As a proof of concept, the point-of-care digital bioanalysis was successfully performed in a hospital setting, using amplification-free multiplex digital RNA detection of SARS-CoV-2, influenza A virus, and influenza B virus. Collectively, COWFISH2 will facilitate versatile and convenient digital bioanalysis in POCT, contributing to the improvement of public health, including the prevention of infectious diseases.

## Highlights

• Developed a portable wide-field fluorescence imaging system for digital bioanalysis
• Developed low-cost and durable consumables for digital bioanalysis
• Developed a stable storage method for the assay reagents used in digital bioanalysis
• Performed a point-of-care digital bioanalysis in a hospital setting

## Graphical abstract

**Figure:**
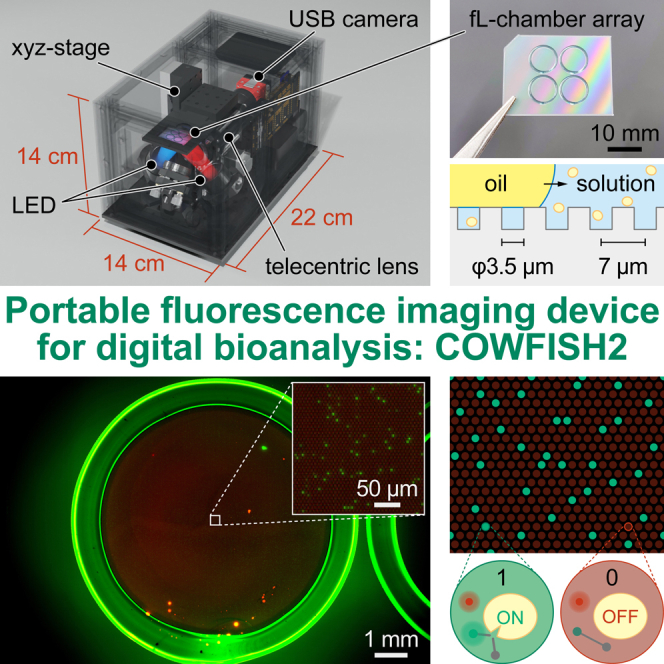

## Introduction

Digital bioanalysis using femtoliter (fL) chamber arrays facilitates the single-molecule analysis of biological reactions with high sensitivity, accuracy, and throughput, enabling the study of rare or heterogeneous biomolecules that are difficult to analyze using conventional bulk methods.^1^^,^^2^ The fL-chamber array can be used to isolate individual molecules and perform various biochemical or biophysical assays, such as DNA/RNA profiling, protein analysis, and drug screening.^3^^,^^4^^,^^5^^,^^6^^,^^7^^,^^8^ The results of these assays can be digitized to provide a variety of insights into biomolecules and even detailed mechanisms related to diseases. Therefore, digital bioanalysis using fL-chamber arrays is a rapidly advancing approach that can potentially revolutionize our understanding of biology and disease and accelerate the development of new therapies and diagnostics.

Owing to the COVID-19 outbreak, the development of a more versatile platform for infectious disease diagnosis is crucial.^9^^,^^10^^,^^11^ In particular, the isolation of positive patients through rapid and accurate diagnosis is essential for infectious disease control^12^; therefore, the development of a rapid, highly sensitive and accurate viral diagnostic platform for point-of-care testing (POCT) is crucial.^13^^,^^14^ Consequently, various microfluidic devices have been developed for nucleic acid and antigen testing.^15^^,^^16^^,^^17^ Of note, diagnostic platforms based on digital bioanalysis, including CRISPR-based amplification-free digital RNA detection (SATORI),^18^ have achieved better performance than existing tests, such as PCR or immunochromatography, in terms of sensitivity, accuracy, or throughput^18^^,^^19^^,^^20^^,^^21^^,^^22^^,^^23^; however, technical challenges remain in realizing POCT, such as the need for a portable detection platform and inexpensive and durable consumables.

In this study, we developed a portable wide-field fL-chamber imaging system (COWFISH2) and low-cost and durable consumables for digital bioanalysis. COWFISH2 demonstrated multiplexed digital detection of the respiratory viral RNA of SARS-CoV-2, influenza A virus (FluA), and influenza B virus (FluB) using CRISPR-Cas13a, validating its utility for point-of-care diagnosis of respiratory viral infections in hospital settings.

## Results

### Development of COWFISH2

**Figure 1:**
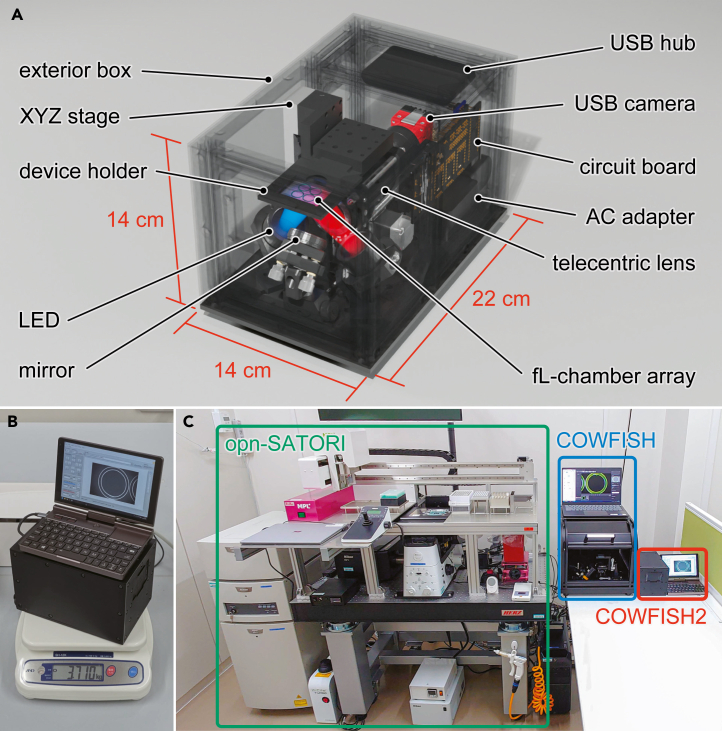
COWFISH2: Portable wide-field fL-chamber imaging system (A) 3D-illustration and (B) photograph of COWFISH2 (size: W14 × D22 × H14 cm, weight: 3.7 kg). (C) Size comparison of opn-SATORI (confocal microscopy), COWFISH, and COWFISH2.

For POCT, we developed COWFISH2, a portable wide-field femtoliter-chamber imaging system by redesigning various electronic controls and optical systems of the previous COWFISH.^24^ COWFISH2 consists of a complementary metal oxide semiconductor (CMOS) camera, telecentric lens, and LED illumination system with the addition of a new 3-axis motorized sample stage (Figure 1A). Regarding POCT, reducing the size of the camera and telecentric lens was the primary challenge, which occupied most of the footprint of the COWFISH. For the CMOS camera, a USB connectable machine vision camera (Alvium 1800 U-2050m, Allied Vision, size: W32 × D38 × H29 mm) mounted with a Sony IMX183 sensor was selected as a commercially available product with a relatively large sensor area (5,496 × 3,672 pixels) and a resolution capable of resolving fL-chamber arrays (pixel size: 2.4 × 2.4 μm), which has a 3.1-fold smaller pixel size and 8.2-fold smaller footprint than the digital single-lens reflection camera used previously (D7500, Nikon, sensor area: 5,568 × 3,712 pixels, pixel size: 4.2 × 4.2 μm, size: W136 × D73 × H104 mm).^24^ To obtain high-resolution and bright fluorescence images, telecentric lenses with high NA should be used, as compared to those previously installed in COWFISH (LSTL20H-F, Myutron, NA: 0.12, size: ϕ61 × L237 mm). Therefore, the following two commercial products were selected as candidates (Figure S1), which have relatively a high NA and small lens barrel size (VTL0714V, Myutron, NA: 0.059–0.073, size: ϕ48 × L122–173 mm; VS-TCT1-65S, VS-technology, NA: 0.11, size: ϕ37 × L107 mm).

**Figure 2:**
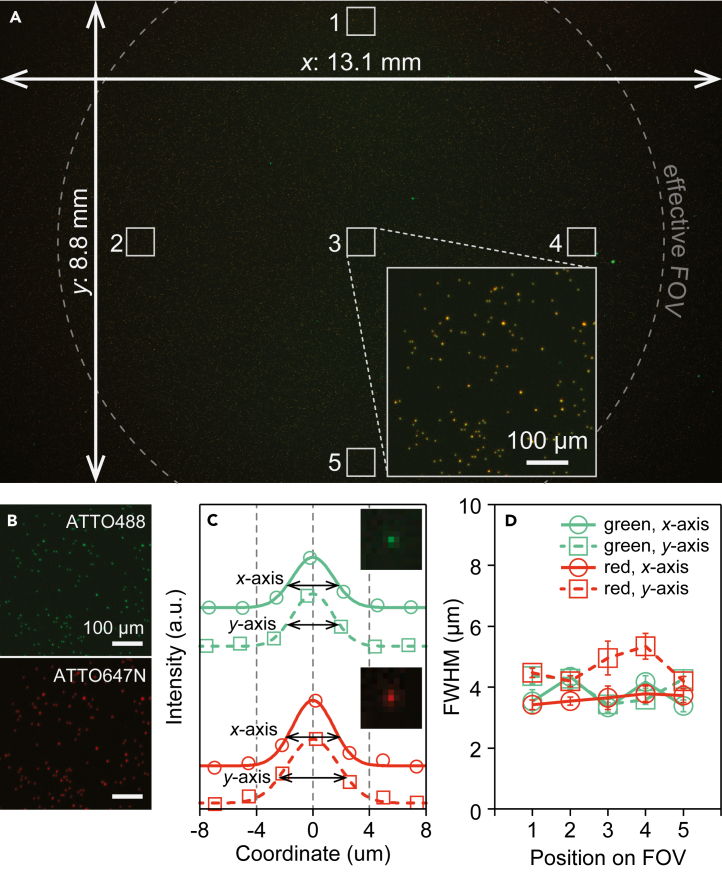
Wide-field fluorescence imaging (A) Fluorescence image of fluorescent magnetic beads (ϕ1 μm). (B) Enlarged view of position 3 in (A). (C) Derived intensity profile. Inset is the representative fluorescence image. (D) FWHM calculated from (C) (*n* = 5 technical replicates).

To assess spatial resolution, fluorescence images of fluorescent magnetic beads of ϕ1 μm labeled with ATTO 488 and ATTO 647N were captured in 20 s (10 s for ex. 470 nm, 10 s for ex. 625 nm). A total of 250 beads were analyzed at five positions (center and four peripheral positions of the effective field of view [FOV]) to determine the *x*- and *y*-intensity profiles, which were then fitted with a Gaussian function to calculate the full width at half maximum (FWHM), a parameter of spatial resolution. The FWHM decreased as the NA values of the telecentric lens increased (Figure S2), and the smallest FWHM at the center of FOV was obtained with the VS-TCT1-65S as 3.3 ± 0.2 and 3.4 ± 0.2 μm for the green channel and 3.7 ± 0.4 and 5.0 ± 0.6 μm for the red channel (mean ± SD for *x*- and *y* axis, respectively), which was nearly constant regardless of image position (Figures 2 and S2; Table S1). One possible reason for the discrepancy between the green and red channels is that red has a longer wavelength than green, which tends to have poorer spatial resolution. Although the spatial resolution between the green and the red channels is different, our results show that VS-TCT1-65S can resolve the fL-chamber arrays with a chamber pitch of 7 μm, as used in this study. In addition, the size of VS-TCT1-65S was 3.5-fold smaller than that previously installed in COWFISH (LSTL20H-F); therefore, we selected VS-TCT1-65S as a telecentric lens for COWFISH2. Reducing the size of the LED illumination system was another challenge. The controller, which occupies most of the LED illumination system, consists of a custom-made electrical board and a smaller holder for commercially available LEDs, newly designed and created, thereby reducing the size of the system by approximately 6.2-fold. The excitation intensity is 1.4 mW/mm^2^ at 470 nm and 1.7 mW/mm^2^ at 625 nm, respectively, which were nearly equal to those of COWFISH.^24^

To expand the versatility, a 3-axis motorized sample stage was developed and implemented to realize multiplex digital bioanalysis using multiple reaction wells in parallel. The 3D position of the motorized stage could also be controlled using the custom-made electrical board described above, allowing multiple assays to be performed on a single array. In COWFISH2, most of the housings and holding jigs were 3D printed with resin to reduce weight. Subsequently, COWFISH2 has a footprint of 14 × 22 cm and a weight of 3.7 kg, including the control PC (Figures 1B and 1C), one-fifth and one-sixth of COWFISH,^24^ which is nearly equivalent to that of commercially available benchtop POCT qPCR instruments (Liat, Roche; ID Now, Abbot; GeneXpert, Beckman Coulter).

### Screening of plastic resin for the femtoliter chamber array

**Figure 3:**
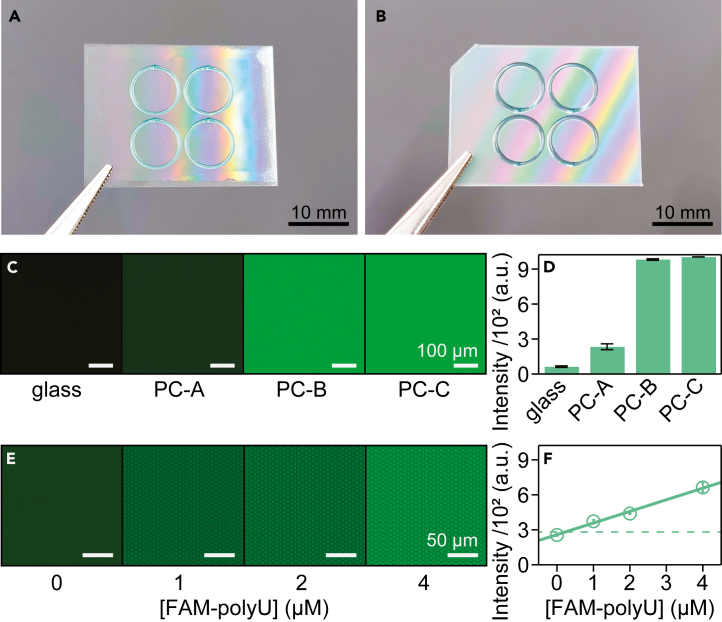
Fluorescence imaging of the fL-chamber array of different materials (A and C) Photographs of the fL-chamber array comprised of glass (A) or PC-A (B). (C) Representative autofluorescence images of fL-chamber arrays made of glass, PC-A, PC-B or PC-C, in the green channel. (D) Autofluorescence intensity measured from (C). (*n* = 3 technical replicates) (E) Fluorescence images of the fL-chambers containing an indicated concentration of FAM-polyU (green). (F) Fluorescence intensity against the concentration of FAM-polyU. The linear regression is represented by a solid line. The value of the blank mean + 3 SD is shown as a dotted line (*n* = 3 technical replicates).

Telecentric lenses enable a greater depth of field and easier focusing but increase the influence of autofluorescence from the fL-chamber array and sample. Subsequently, arrays comprised of conventional polycarbonate, which are used in the production of compact disks, have strong autofluorescence in the green channel, complicating their use for digital bioanalysis when using the COWFISH,^24^ that is, a glass array was used instead of a plastic array due to low autofluorescence.^18^^,^^19^^,^^25^ In this study, by using an advanced polycarbonate for optical components such as light guide plates (PC-A), the autofluorescence of the fL-chamber array was reduced to less than one-fourth for the green channel (Figures 3A–3D), allowing an limit of detection (LoD) of 0.24 ± 0.13 μM (mean ± SD) for FAM-polyU (Figures 3E and 3F). Because conventional digital bioanalysis detects fluorescent products of 1–4 μM, the LoD values of COWFISH2 with a plastic fL-chamber are sufficient to demonstrate digital bioanalysis, contributing to a 25th cost reduction of the fL-chamber array. Hereafter, we used a plastic fL-chamber array comprised of PC-A for digital bioanalysis.

### Demonstration of digital bioanalysis: SATORI assay

**Figure 4:**
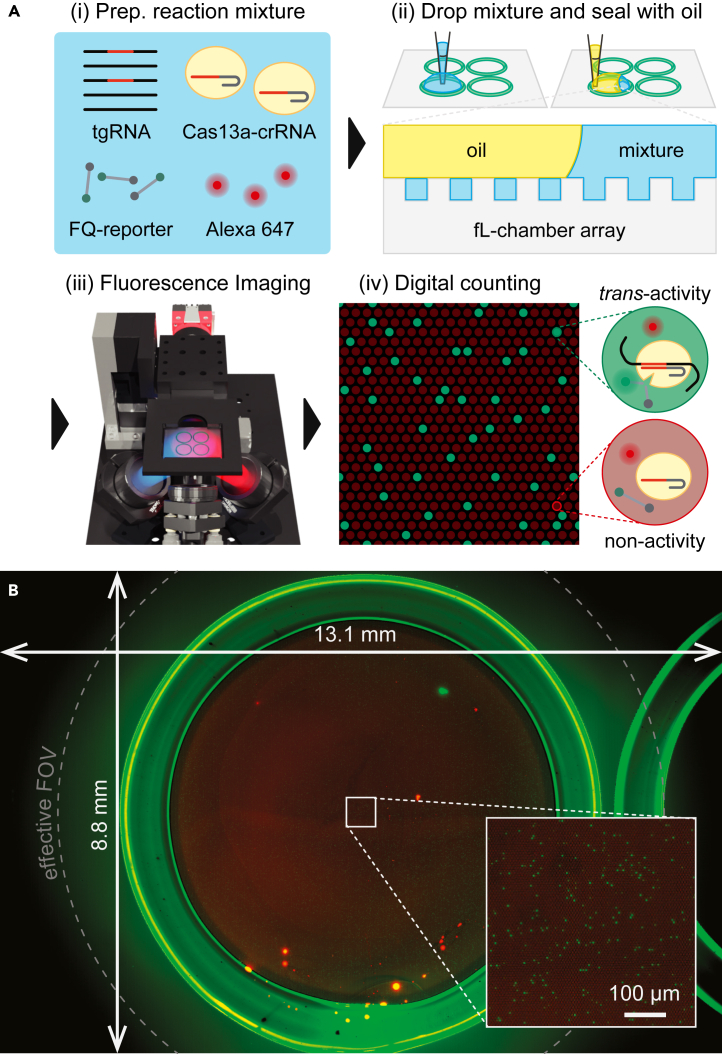
Digital viral RNA detection by SATORI assay (A) Schematic illustration. Upon binding to viral RNA, the Cas13a-crRNA complex cleaved the FQ-reporters, leading to increased fluorescence in the fL-chamber. (B) Fluorescence image captured by COWFISH2. The green circle represents the enclosure of ϕ7 mm, which contains approximately 900,000 fL-chambers. The inset is the zoom-up.

**Figure 5:**
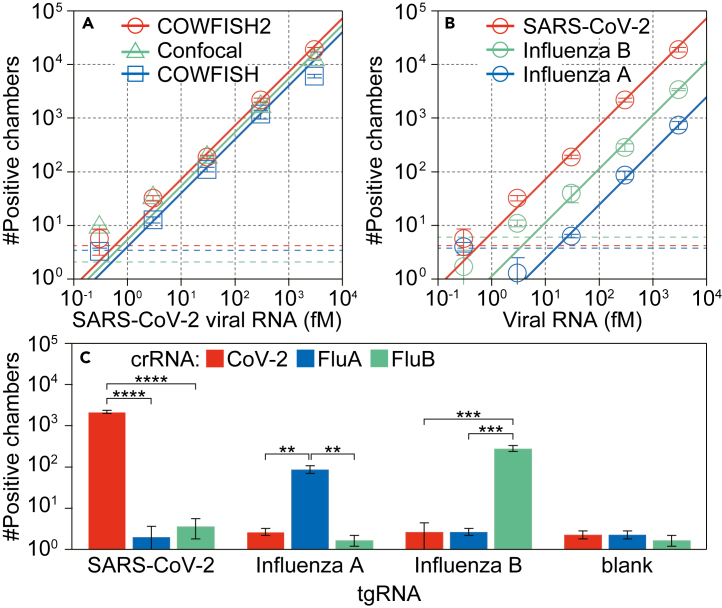
Digital counting of viral RNAs (A) Number of positive chambers obtained with COWFISH2 (red), COWFISH (blue) or confocal microscopy (green). The linear regressions are represented by solid lines. The values of the blank mean +3 SD are shown as dotted lines (*n* = 3 technical replicates). (B) Number of positive chambers obtained using COWFISH2 for SARS-CoV-2 (red), FluA (blue), or FluB (green). The linear regressions are represented by solid lines. The values of the blank mean + 3 SD are shown as dotted lines (*n* = 3 technical replicates). (C) Number of positive chambers obtained using crRNA for SARS-CoV-2 (red), FluA (blue) or FluB (green). The concentration of tgRNA was 300 fM. Asterisks represent the significant differences determined by unpaired two-tailed Student’s t test with equal variance (∗∗: *p* < 0.01, ∗∗∗: *p* < 0.001, and ∗∗∗∗: *p* < 0.0001).

CRISPR-Cas has recently garnered interest as a new genetic test for the diagnosis of viral infections and underlying diseases,^17^^,^^26^ and some platforms that use Cas12a/13a have been developed based on digital bioanalysis for the highly sensitive and rapid detection of viral genomes.^18^^,^^19^^,^^27^^,^^28^^,^^29^ As a proof of concept for digital bioanalysis, the feasibility of COWFISH2 was investigated for digital viral RNA detection using CRISPR-Cas13a (SATORI) and compared with the conventional method employing COWFISH and confocal fluorescence microscopy (Figures 4 and 5). Considering the recent global pandemic, the viral RNA genomes derived from the respiratory infection viruses (SARS-CoV-2, FluA, and FluB) were selected as targets for the SATORI assay. In the SATORI assay (Figure 4A), a mixture of the viral RNA genome (tgRNA), LtrCas13a complexed with crRNA, and fluorescent reporters (FAM-based: FQ-reporter) was dropped onto the arrays and encapsulated into the fL-chambers by dropping oil. After incubation for a few minutes, the fL-chambers containing Cas13a-crRNA-tgRNA complexes exhibited fluorescence signals resulting from the cleavage of the FQ reporters. These positive chambers were quantitatively counted from the fluorescence images obtained (Figure 4B). Therefore, quantifying the amount of viral RNA in the samples was possible.

Using COWFISH2, the entire reaction well (ϕ7 mm, approximately 900,000 fL-chamber arrays) was captured as a single fluorescence image in 11 s (10 s for ex. 470 nm, 1 s for ex. 625 nm), whereas COWFISH with glass arrays requires 20 s for image acquisition. Across a broad range of viral RNA concentrations (approximately 1 fM–3 pM), the number of positive chambers determined by COWFISH2 analysis increased linearly, as observed by COWFISH^24^ or confocal microscopy (Figure 5A). The LoD value for SARS-CoV-2 obtained using COWFISH2 (0.58 ± 0.05 fM, mean ± SD) was nearly identical to 0.89 ± 0.15 or 0.39 ± 0.06 fM (mean ± SD) obtained using COWFISH or confocal microscopy, respectively. This result is demonstrating that COWFISH2 can be used for viral RNA quantification, thus, for digital bioanalysis with plastic fL-chamber arrays.

For FluA and FluB, the LoD values were obtained using COWFISH2 as 15 ± 2 and 5.4 ± 0.2 fM (mean ± SD) (Figure 5B). In addition, the use of screened crRNA that is not complementary to tgRNA resulted in a negligible number of positive chambers (Figure 5C), comparable to the background level (the number of positive chambers was lower than 10). This finding confirms the absence of cross-reactivity in the SATORI assay and suggests that COWFISH2 can potentially be expanded for multiplex detection, targeting not only SARS-CoV-2 but also FluA and FluB.

### Assay reagent storage for SATORI

**Figure 6:**
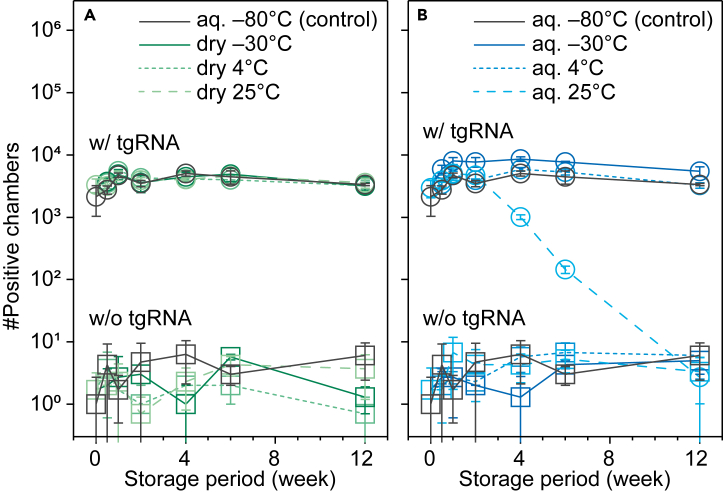
Storage method for SATORI reagents (A and B) Number of positive chambers obtained using lyophilized (A) or solution SATORI reagents (B) with (circle) or without tgRNA (square). (*n* = 3 technical replicates). The concentration of tgRNA (N-gene of SARS-CoV-2) was 300 fM.

The storage conditions for assay reagents are crucial in clinical settings. Most commercial reagents used in the diagnosis of COVID-19 as POCT are stored at 4°C for over one month and do not require large, specialized storage facilities such as deep freezers, eliminating the dependence on cold chains. Therefore, we aimed to develop a simple method without cold chains for long-term storage of the reagents used in the SATORI assay. The storage method was optimized using lyophilization, a common method used to store protein reagents including Cas proteins for long periods of time.^30^^,^^31^ For comparison, both solution and lyophilized SATORI reagents, including Cas13a, crRNA, and FQ-reporters, were prepared and stored at ˗30, 4, or 25°C for three months. Using SATORI, the condition of each reagent was evaluated over time (Figure 6), showing that the number of positive chambers, which reflects the active fraction of Cas13a, did not change significantly with lyophilization for three months, whereas that with solution storage at 25°C substantially decreased to the background level after one month, indicating that lyophilization storage is better for the SATORI assay.

### Clinical validation as POCT

**Figure 7:**
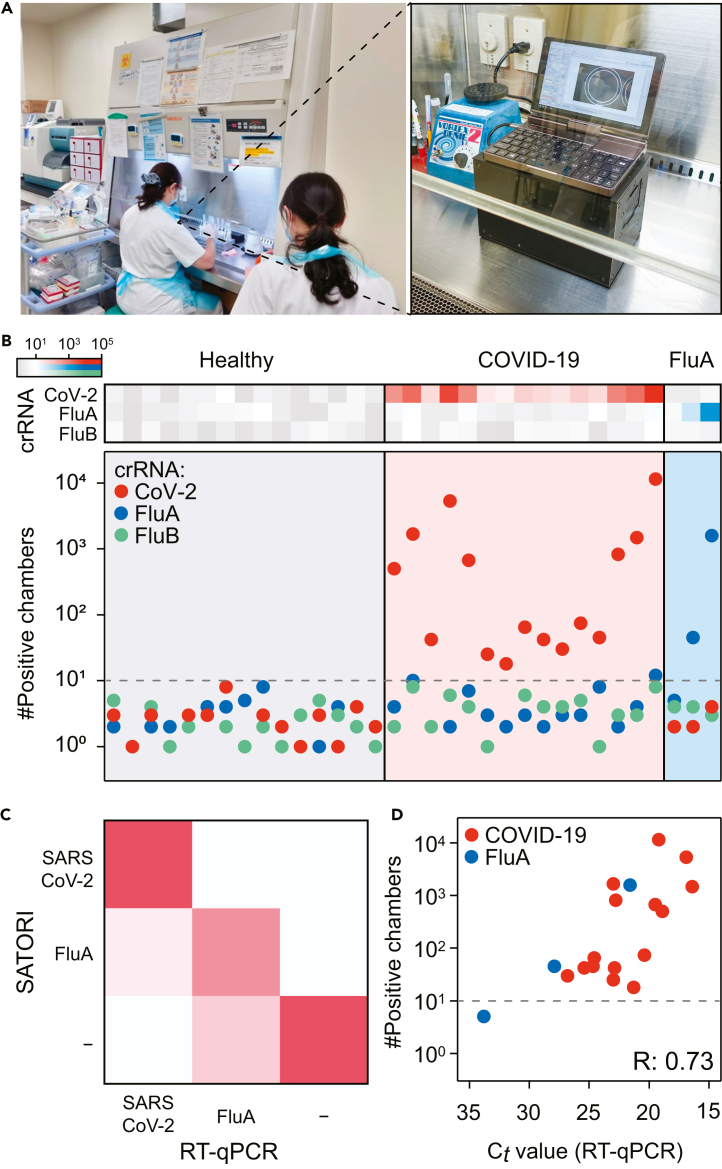
Clinical validation as POCT (A) Photographs of COWFISH2 equipped in the safety cabinet at the hospital of Tokyo Metropolitan Institute for Geriatrics and Gerontology. (B) Multiplexed detection of SARS-CoV-2/FluA/FluB in clinical specimens. The positive threshold is indicated by a dashed line. (C) Comparison of SATORI and RT-qPCR results in viral genome detection. (D) Comparison of the number of positive chambers for SATORI and Ct value for RT-qPCR.

Recently, genetic testing, that is, PCR, for FluA and FluB, in addition to that for SARS-CoV-2, has been performed simultaneously to diagnose respiratory infections. Such a diagnosis has also confirmed multiple infections not only with SARS-CoV-2 but also with FluA and FluB, and the identification of these infections is crucial for protection against infectious diseases. Therefore, as a clinical validation of SATORI using COWFISH2 as a POCT, multiplex digital RNA detection was performed for SARS-CoV-2, FluA, and FluB in a clinical setting (Figure 7). The footprint of COWFISH2 is minimal, but sufficient to be collocated with conventional clinical laboratory equipment, and its installation is easy in clinical settings (Figure 7A). The proof-of-concept using a clinical specimen showed that the multiplex detection of three respiratory viruses, SARS-CoV-2/FluA/FluB, was completed in approximately 20 min. The LoD obtained using clinical specimens, referred to as the clinical LoD, was 6.7 fM for SARS-CoV-2 (Figure S3). This was higher than the analytical LoD of 0.58 fM obtained using viral genomes purified from virus-infected cultured cell lines (Figure 5C). Several POCT diagnostic platforms have reported that the LoD is affected when clinical specimens are used,^32^^,^^33^ suggesting a similar influence is present in SATORI with COWFISH2. The sensitivity and specificity of multiplex detection were 94% (17/18) and 98% (79/81), respectively, under the same positive criteria previously used for SATORI^24^ (number of positive chambers ≥10) (Figures 7B and 7C). The results were highly correlated with quantitative reverse-transcription PCR (RT-qPCR) (Figure 7D), demonstrating that digital bioanalysis with COWFISH2 enables the rapid and accurate point-of-care diagnosis of infectious diseases in clinical settings.

## Discussion

We developed a portable and wide-field fluorescence imaging system called COWFISH2 that captures fluorescence images of sub-million fL-chambers with a spatial resolution of approximately 3 μm. COWFISH2 was constructed using simple and inexpensive components, resulting in a reduction in size to W14 × D22 × H14 cm and a reduction in cost to US$5,424 (Table S2), which is approximately 20% and 64% of COWFISH,^24^ respectively. Owing to its small and lightweight design, we successfully transported COWFISH2 in a backpack from Japan to Singapore in June 2023 and performed the SATORI assay for SARS-CoV-2 detection, demonstrating its versatility and portability. In addition, the cost of consumables, such as fL-chamber arrays, was reduced, and the shelf life of the assay reagents was improved, resulting in simplified storage methods. As a proof-of-concept, digital bioanalysis using COWFISH2 demonstrated the multiplex detection of respiratory viral RNAs in clinical settings with high sensitivity and specificity equivalent to conventional setups, contributing to the rapid and accurate point-of-care diagnosis of respiratory viral infections.^34^ In summary, these advantages support the application of COWFISH2 for rapid and cost-effective digital bioanalysis for POCT, and we envision it as a versatile platform for liquid biopsy in the future.

### Limitations of the study

In this article, a proof-of-concept experiment was performed as clinical validation using the minimum required clinical specimens. The performance of the device developed in this study, COWFISH2, could be evaluated and improved through further validation experiments using a large number and variety of clinical specimens.

## Resource availability

### Lead contact

Further information and requests for resources and reagents should be directed to and will be fulfilled by the lead contact, Rikiya Watanabe (rikiya.watanabe@riken.jp).

### Materials availability

This study did not generate new unique reagents.

### Data and code availability

• All data reported in this paper will be shared by the lead contact upon request.
• This paper does not report original code.
• Any additional information required to reanalyze the data reported in this paper is available from the lead contact upon request.

## Acknowledgments

We thank all members of the Watanabe, Toyoda, and Noda laboratories for their constructive discussions, Mr. K. Sugiyama for the fabrication of the plastic fL-chamber array, and the Support Unit for Bio-Material Analysis of Research Resources Division and the Advanced Manufacturing Support Team at RIKEN for technical assistance. This work was supported by JST CREST (JPMJCR19H5), 10.13039/100009619AMED (JP22he0422018), and a Grant-in-Aid for Transformative Research Areas A (20H05931) to R.W.; 10.13039/100009619AMED (JP22fk0108542) to M.T. and R.W.; JSPS Grant-in-Aid for Scientific Research A (21H04645) to T.N. and R.W.; and JST CREST (JPMJCR20HA) and JSPS Core-to-Core program A to T.N.

## Author contributions

T.I. and R.W. designed the experiments; H.S., M.Y., A.M., and M.N. performed sample preparation; T.I., J.A., and R.W. developed COWFISH2; T.I. performed the digital bioanalysis; Y.K. supported the clinical trials; M.T., T.N., and R.W. supervised the study; T.I. and R.W. wrote the manuscript.

## Declaration of interests

The authors declare no competing interests.

## STAR★Methods

### Key resources table

**Table:** 

REAGENT or RESOURCE	SOURCE	IDENTIFIER
**Bacterial and virus strains**		

*Escherichia col*i Rosetta 2 (DE3) Competent Cells	Sigma-Aldrich	Cat#71397

**Biological samples**		

FluA genome RNA	In-house	N/A
FluB genome RNA	In-house	N/A
LtrCas13a protein	In-house	(Shinoda et al.^18^)
SARS-CoV-2 genome RNA	In-house	N/A

**Chemicals, peptides, and recombinant proteins**		

Alexa Fluor 647 C2 maleimide	Thermo Fisher Scientific	A20347
ATTO 488 biotin	ATTO-TEC	AD488-71
ATTO 647N biotin	ATTO-TEC	AD647*N*-71
Dulbecco’s Modified Eagle’s Medium	Sigma-Aldrich	N/A
Fomblin oil	Solvay	Y LVAC 25/6
Minimal Essential Medium	Thermo Fisher Scientific	N/A
MyOneT1 streptavidin	Invitrogen	Cat#65601
newborn calf serum	Thermo Fisher Scientific	Cas#16010-159

**Critical commercial assays**		

Ampdirect™ 2019-nCoV Detection Kit	SHIMADZU	Cat#227-20000-21
QIAamp Viral RNA Mini Kit	Qiagen	Cat#52904
RNeasy kit	Qiagen	N/A
TRexGene SARS-CoV-2&FluA/B Detection Kit	Toyobo	QSTRG-201

**Oligonucleotides**		

crRNA for FluA	GeneDesign	N/A
crRNA for FluB	GeneDesign	N/A
crRNA for SARS-CoV-2	GeneDesign	N/A
FAM-polyU	Hokkaido System Science	N/A
FQ-reporter	IDT	N/A

**Recombinant DNA**		

pET-LtrCas13a plasmid	In-house	(Shinoda et al.^18^)

**Software and algorithms**		

Igor Pro	WaveMetrics	https://www.wavemetrics.com/
ImageJ	NIH ImageJ	https://imagej.net/software/imagej/
LabVIEW	National Instruments	https://www.ni.com/
Microsoft Excel	Microsoft	https://www.microsoft.com/

### Experimental model and study participant details

#### VeroE6 cells and MDCK cells

VeroE6/TMPRSS2 cells were cultured in Dulbecco’s Modified Eagle’s Medium (Sigma-Aldrich) containing 10% fetal calf serum and 1% penicillin/streptomycin (PS) at 37°C under 5% CO2. MDCK cells were grown in Minimal Essential Medium (Thermo Fisher Scientific) containing 5% newborn calf serum (16010-159, Thermo Fisher Scientific) and 1% PS.

#### Patient samples

The patient samples were collected from fifteen SARS-CoV-2 (saliva) and three FluA (nasal/nasopharyngeal swab) patients and fifteen healthy individuals (saliva) at the Tokyo Metropolitan Institute for Geriatrics and Gerontology according to procedures approved by the Ethics Review Board (R22-059). The information of samples such as the age, gender are not available due to ethical and privacy restriction. However, we don’t anticipate the age or gender of the participants to have any influence on the results. RNA was extracted from the samples using a QIAamp Viral RNA Mini Kit automated on a QIAcube (Qiagen) according to the instructions of the manufacturer. The RNA was eluted in nuclease-free water. Reverse transcription-quantitative PCR (RT-qPCR) was performed using the extracted viral RNA with QuantStudio 3 (Thermo Fisher Scientific) and Ampdirect 2019-nCoV Detection Kit for SARS-CoV-2 (SHIMADZU) or TRexGene SARS-CoV-2&FluA/B Detection Kit for FluA and FluB (Toyobo). The research was performed in accordance with the relevant guidelines and regulations, and the informed consent was obtained from all participants.

### Method details

#### Configuration of COWFISH2

COWFISH2 was constructed using a monochrome complementary metal oxide semiconductor (CMOS) camera (1800U-2050m, Allied Vision) and low-distortion 1× telecentric lens (VS-TCT1-65, VS Technology) (Figure 1). The camera has a 13.1 × 8.8 mm back-illuminated commercial CMOS sensor with a sensor pixel pitch of 2.4 μm/pixel (IMX 183, Sony). A telecentric lens with an image circle of ϕ11 mm, a working distance of 66 mm, and a numerical aperture (NA) of 0.11 was mounted directly on the camera via a C-mount. To observe the horizontally placed samples from below, the optical path was bent at a right angle using a mirror placed 45° under the sample. A sample holder was mounted on a custom 3-axis motorized stage (XEG25/XYEG40, MISUMI; PKP213D05A, Oriental Motor) to adjust the z-position for image focusing and the xy-position for centering each reaction well.

Two LED light sources (M470D4 and M625D3, Thorlabs) with center wavelengths of 470 and 625 nm, respectively, were installed for fluorescence excitation. Light from these LED units was collimated with condenser lens (ACL12708U-A, Thorlabs, ϕ1/2″, f = 8 mm, NA: 0.78) and filtered using a single-band excitation filter for 470 (ET480/20x, Chroma, ϕ1″) or 625 nm (ZET635/20x, Chroma, ϕ1″). An excitation filter was attached to the LED mount head using a lens tube (SM1L10E, Thorlabs), and a condenser lens was placed between them using a lens holder (SM1A6T, Thorlabs). These LED units were placed under the sample holder at an angle of approximately 35° and distance of approximately 8 cm to illuminate an area with a ϕ of 1.8 cm. The light intensity in the sample plane was 1.4 mW/mm^2^ for 470 nm and 1.7 mW/mm^2^ for 625 nm. For fluorescence imaging, a quad-band pass emission filter (89402m, Chroma, ϕ1″) was inserted in front of the telecentric lens. The camera, lens, stage, and LEDs were fixed on a 14 × 22 cm breadboard using DIY components provided by Thorlabs. The optics were covered with black resin frames fabricated using a masked stereolithography apparatus 3D printer (Saturn2, ELEGOO) for shielding from external light during fluorescence imaging.

The LEDs and 3-axis motorized stage were controlled by LED drivers (RCD-24-1.00, RECOM) and stepper motor drivers (Pololu-1182, Pololu) using a microcontroller (JP-EL-CB-005, ELEGOO, compatible Arduino Nano V3.0) on a custom electrical circuit board. The drivers were controlled by a custom-made LabVIEW software.

#### Fabrication of fL-chamber array comprising polycarbonate

The original mold was prepared by photolithography using a 3D drawing system (DWL66+, Heidelberg Instruments), and a Ni replica was fabricated by electrocasting. The fL-camber array was fabricated from three types of polycarbonate (PC) resin; PC-A (SD POLYCA TR1801A, Sumika Polycarbonate), PC-B (Jupilon H-4000, Mitsubishi Engineering-Plastics), and PC-C (TARFLON MD1500, Idemitsu Kosan); using a molding machine (SD40ER, Sumitomo Heavy Industries) with the above mold as previously reported.^19^ A UV-curable acrylic resin (5 × 649H, CHEMITECH) and dispensing robot (SHOTmini 200SX SM200S, Musashi Engineering) were used to fabricate four ring-shaped enclosures as reaction wells with 7-mm inner diameter on the 32 × 24 mm microchip. The volume of a single chamber was 33.7 fL with a diameter and depth of 3.5 and 3.5 μm, respectively.

#### Preparation of Cas13a

The Cas13a from Leptotrichia trevisanii (LtrCas13a) was expressed and purified as previously reported.^24^ For the expression of LtrCas13a, Escherichia coli Rosetta 2 (DE3) was transformed with the pET-LtrCas13a plasmid, and the cells were cultured in a 2.5-L LB medium containing kanamycin. When the OD600 values reached 0.6–1.0, the cells were cooled on ice for 10 min and further cultured at 20°C for 20 h with 0.1 mM IPTG. Bacterial cells were collected by centrifugation, suspended in 40 mL of buffer A (20 mM Tris-HCl [pH 8.0], 1 M NaCl, 20 mM imidazole, 3 mM β-mercaptoethanol, and 1 mM phenylmethylsulfonyl fluoride), and lysed via sonication (Q500, QSONICA). After centrifugation at 15,000 rpm for 20 min, the supernatant was incubated with Ni-NTA agarose (Qiagen) at 4°C for 1 h. The mixture was then transferred to an Econo column (Bio-Rad). The resin was washed with buffer B (20 mM Tris-HCl [pH 8.0], 0.3 M NaCl, 20 mM imidazole, and 3 mM β-mercaptoethanol), and the protein was eluted with buffer C (20 mM Tris-HCl [pH 8.0], 0.3 M NaCl, 300 mM imidazole, and 3 mM β-mercaptoethanol). The protein was then loaded onto a HiTrap SP HP column (Cytiva) equilibrated with buffer D (50 mM HEPES-KOH [pH 7.5], 0.3 M NaCl, and 0.5 mM TCEP). The protein was eluted using a linear gradient from 0.3 to 2.0 M NaCl over seven column volumes. It was further purified through size exclusion chromatography (Enrich SEC 650, Bio-Rad) with buffer E (50 mM HEPES-KOH [pH 7.5], 0.5 M NaCl, and 0.5 mM TCEP).

#### Preparation of crRNA, FQ-reporter, and viral RNA

Chemical synthesis crRNAs targeting the N-gene of SARS-CoV-2 (GGAUUUAGAGUACCCCAAAAAUGAAGGGGACUAAAACAAGGUCUUCCUUGCCAUGUUGAGUGAGAGCGG), the NP-gene of FluA (GGAUUUAGAGUACCCCAAAAAUGAAGGGGACUAAAACAUGAGUAAUGAAGGGUCUUAUUUCUUCGGAGA), or that of FluB (GGAUUUAGAGUACCCCAAAAAUGAAGGGGACUAAAACUUUUGGGCUCCA8AUGACCAGAUCUGGGGGGAA) were purchased from GeneDesign. The FQ-reporter (FAM/rUrUrUrUrU/3lABkFQ) and FAM-polyU (FAM/rUrUrUrUrU) were obtained from IDT and Hokkaido System Science, respectively.

Viral RNA from SARS-CoV-2, FluA, and FluB was prepared as follows: SARS-CoV-2/Hu/DP/Kng/19–027 (Wuhan lineage), FluA (A/California/04/2009(H1N1)) and FluB (B/Massachusetts/02/2012) were propagated in VeroE6/TMPRSS2 cells (JCRB 1819) (for SARS-CoV-2) or Madin-Darby canine kidney (MDCK) cells (for FluA and FluB). Viral supernatants were collected 2 days after infection, and viral RNA was purified using an RNeasy kit (Qiagen). The concentration of the viral RNA was determined from the A260 value using a NanoDrop spectrophotometer (Nanodrop, Themo Fisher Scientific).

#### SATORI assay

SATORI assay was performed as previously reported.^24^ The assay solution (solution A) for a single assay was prepared as follows: To prepare Cas13a-crRNA complexes, a mixture of 0.6 μL of Cas13a (20 μM), 1.2 μL of crRNA (2.5 μM), and 2.2 μL of buffer F (20 mM HEPES-KOH [pH 6.8], 60 mM NaCl, 6 mM MgCl2, and 50 μM Triton X-100) was incubated at 37°C for 10 min. Next, 4.0 μL of Cas13a-crRNA solution was mixed with 6.0 μL of buffer G (20 mM HEPES-KOH [pH 7.5], 100 mM NaCl, 10 mM MgCl2, 50 μM Triton X-100, 40 μM FQ-reporter), and stored at the indicated temperature (−80, −30, 4 or 25°C) until use.

For SATORI assay, 10 μL of solution A was mixed with 10 μL of buffer H (20 mM HEPES-KOH [pH 7.5], 100 mM NaCl, 10 mM MgCl2, 50 μM Triton X-100, and 120 μM Alexa Fluor 647 C2 maleimide (Thermo Fisher Scientific)), and was then added to 100 μL of target RNA solution. After 1 min of incubation, 105 μL of the mixture was dropped onto the fL-chamber array. Of the 105-μL solution on the array, 95 μL was removed, and 50 μL of Fomblin oil (Y LVAC 25/6, Solvay) was dropped to seal the fL-chamber. Excess solution A and Fomblin oil remaining on the array were removed. After 3 min of incubation, which was optimized in our previous study,^19^ fluorescence images were captured using COWFISH2, COWFISH, or confocal microscopy with a 20× objective lens (NA: 0.75), 488 and 640 nm lasers, and a motorized 2-axis scanning stage (A1HD25, Nikon). For COWFISH2 or COWFISH, fluorescence images were acquired for 11 s (10 s for ex. 470 nm, 1 s for ex. 625 nm) or 20 s (10 s for ex. 470 nm, 10 s for ex. 625 nm), respectively. For confocal microscopy, fluorescence tiling images with 488 and 640 nm lasers were captured for approximately 200 s with 64-stage scanning. The research was also performed in accordance with the relevant guidelines and regulations.

#### Lyophilization

For the single SATORI assay, 10 μL of solution A was flash frozen in liquid nitrogen and lyophilized overnight using a freeze-drying machine (FDU-830, EYELA). The lyophilized sample was stored at the indicated temperature (−30, 4 or 25°C) until use. Before starting SATORI assay, the lyophilized sample was mixed with 10 μL of nuclease-free water and returned to solution A.

#### Image acquisition

The considerable depth of field of the telecentric lens simplifies adjusting and holding the sample in focus with COWFISH2. A low chromatic aberration of lens in COWFISH2 makes it possible to acquire the green and red fluorescence images at the same z-position. Fluorescence images were captured in a 10-bit TIFF format using custom-made LabVIEW software. The pixel value of grayscale images was defined as fluorescence intensity.

#### Evaluation of pixel size and spatial resolution

Bright-field images of a stage micrometer (OBM1/100SQ, Olympus) with lines at 10-μm intervals were captured at the center and four peripheral positions of the effective field of view (FOV). By dividing the distance of 200 μm by the number of pixels on the image, the pixel sizes were calculated as 2.4 μm for both the x- and y axis at five positions (Figure S1; Table S1).

The fluorescent beads were prepared by mixing 10 mg/mL (approximately 109 beads/μL) magnetic beads (MyOneT1, Invitrogen, ϕ1 μm) with ATTO 488 biotin and ATTO 647N biotin (ATTO-TEC), incubating at 25°C for 10 min, washing with buffer I (20 mM HEPES-KOH [pH 7.5], 100 mM NaCl, 10 mM MgCl2, 500 μM Triton X-100) three times, and adjusting the concentration to 0.1 μg/mL (approximately 104 beads/μL) with buffer I. The fluorescent beads were then immobilized on the surface of a 32 × 24 mm cover glass (No. 1, Matsunami) via non-specific interaction. The fluorescence images were captured for 20 s (10 s for ex. 470 nm, 10 s for ex. 625 nm) at the center and four peripheral positions of the effective FOV.

#### Data analysis for the SATORI assay

The 16- or 10- bit TIFF images obtained by COWFISH^24^ or COWFISH2, respectively, were analyzed using ImageJ Software as follows: Fluorescence images were processed by removing the background signal with the Subtract Background command of ImageJ (parameter: 31-pixel rolling ball radius), followed by the Scale command (parameters: a scale factor of 2.0 for both the x- and y axis, and bilinear interpolation) of ImageJ to improve the region of interest (ROI) recognition of adjacent chambers in the subsequent analysis. The green image was then binarized using an intensity of 9,000 for COWFISH or 120 for COWFISH2 as the thresholds. To determine the positive chambers and obtain ROI information, the binarized images were processed using the Analyze Particles command of ImageJ (parameters: size 7–50 μm2, circularity 0.8–1.0). Under this criterion, some dust on the substrate can be detected, resulting in false positives. Because most dust shows a strong fluorescence signal in both the green and red images, the fluorescence intensity of the red image in the ROI was used to suppress the counting of false-positive chambers. The ratio of the fluorescence intensity in the green and red images (red/green ratio) was calculated, and ROIs with a red/green ratio greater than 0.3 were excluded as false-positive chambers.

The 12-bit TIFF images (red and green) obtained by confocal microscopy were analyzed using NIS-Elements software (Nikon), as follows: The green images were processed using the NIS-Elements Object Count command (parameters: diameter: 3–8 μm, circularity: 0.8–1.0, threshold: 3,000) to determine the positive chambers and obtain their ROI information. Similar to COWFISH, the fluorescence intensity of the red image in the ROI was used to suppress the counting of false-positive chambers. ROIs with a red/green ratio greater than 0.5 were excluded as false-positive chambers.

The analytical limit of detection (LoD) was defined as follows: the number of positive chambers obtained with different concentrations of the target RNA (tgRNA) was fitted to a linear curve. The mean +3 SD value for the number of positive chambers obtained without tgRNA was determined, and the crossing point of the linear curve and the mean +3 SD value were then determined. The concentration corresponding to the crossing point was defined as the LoD value.

### Quantification and statistical analysis

Data in Figures 2, 3, 5, 6, 7, S2, and S3 were processed and visualized using Microsoft Excel and Igor Pro. The data are presented as mean ± SD. Significant differences between two groups were determined by unpaired two-tailed Student’s t test with equal variance. Asterisks represent the significant differences: ∗∗: *p* < 0.01, ∗∗∗: *p* < 0.001, and ∗∗∗∗: *p* < 0.0001.
