# Supplementary material for: Portable wide-field femtoliter-chamber imaging system for point-of-care digital bioanalysis
Source: iScience. 2024 Sep 1;27(9):110868. doi: 10.1016/j.isci.2024.110868 (PMC11416649; doi:10.1016/j.isci.2024.110868)
Supplement: Document S1. Figures S1–S3, Tables S1, and S2 [file mmc1.pdf]

iScience, Volume *27*

Supplemental information

Portable wide-field femtoliter-chamber imaging system for point-of-care digital bioanalysis

Tatsuya Iida, Jun Ando, Mami Yoshimura, Asami Makino, Masahiro Nakano, Yasushi Kogo, Hajime Shinoda, Masashi Toyoda, Takeshi Noda, and Rikiya Watanabe

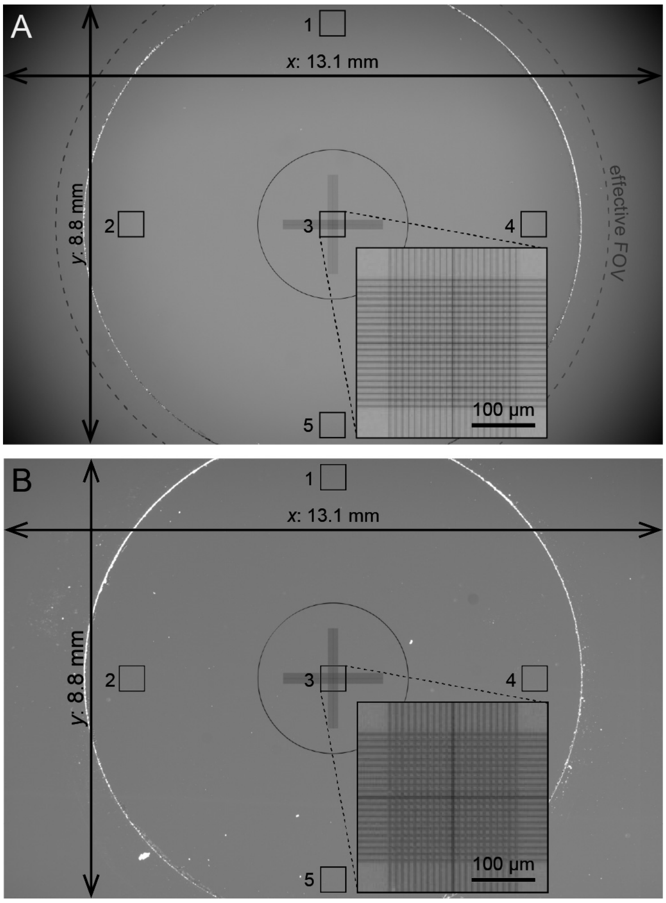

Fig. S1. Bright-field imaging with small telecentric lenses, related to Figure 1

Bright-field image of micrometer observed with VS-TCT1-65S (A) and VTL0714V (B). Line interval is 10 µm. Inset is the zoom-up.

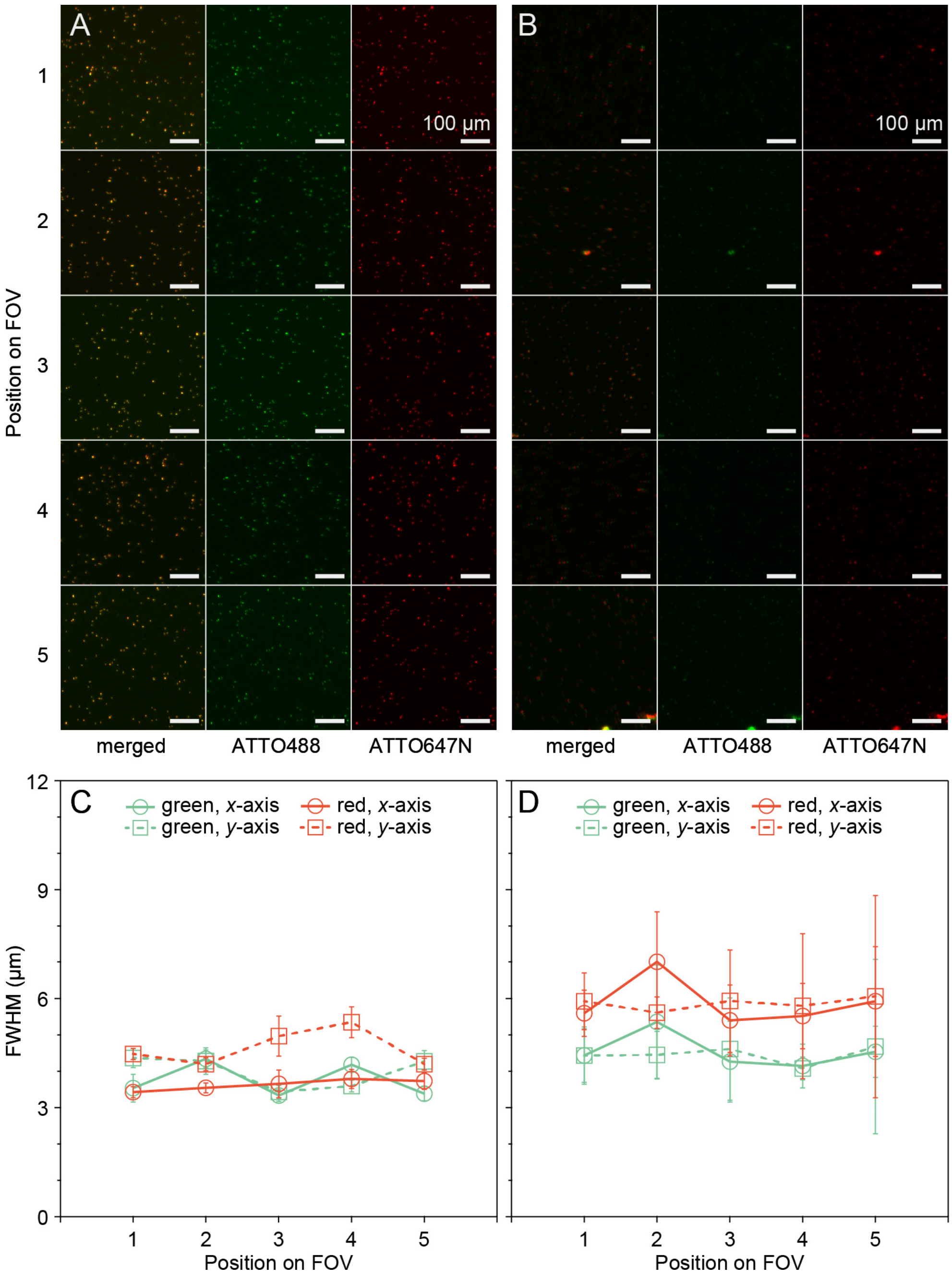

Fig. S2. Two-color fluorescence imaging with small telecentric lenses, related to Figure 2

Fluorescence image of magnetic beads (φ1 µm) labeled with ATTO 488 (green) and ATTO 647N (red) observed with VS-TCT1-65S (A) and VTL0714V (B) at five positions on FOV as shown in Fig. 2. (C, D) FWHM analyzed at five positions on FOV (n = 5 technical replicates).

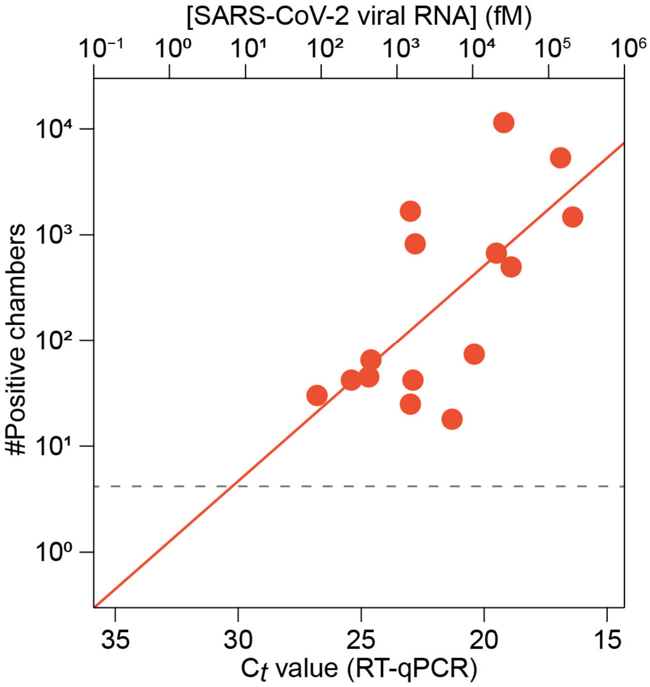

Fig. S3. The limit-of-detection (LoD) for clinical specimens of SARS-CoV-2 patients, related to Figure 7

Comparison of the number of positive chambers for SATORI with Ct value and viral RNA concentration for RT-qPCR. The linear regression is represented by solid line. The values of the blank mean + 3 SD are shown as dotted lines. The clinical LoD is defined from the intersection of the solid and dotted lines, and obtained as Ct 30.3 and 6.7 fM.

Table S1. Pixel size and FWHM, related to Figure 1 and 2

Lens	Position	Pixel size (µm)		FWHM (µm, mean ± SD, n = 50)			
				Green fluorescence		Red fluorescence	
		x-axis	y-axis	x-axis	y-axis	x-axis	y-axis
VS-TCT1-65S (COWFISH2)	1. Top	2.40	2.40	3.53 ± 0.38	4.36 ± 0.26	3.42 ± 0.15	4.47 ± 0.16
	2. Left	2.39	2.40	4.34 ± 0.25	4.28 ± 0.36	3.54 ± 0.14	4.19 ± 0.18
	3. Center	2.40	2.40	3.33 ± 0.16	3.43 ± 0.17	3.65 ± 0.38	4.96 ± 0.55
	4. Right	2.40	2.40	4.17 ± 0.20	3.59 ± 0.16	3.78 ± 0.26	5.34 ± 0.43
	5. Bottom	2.38	2.39	3.38 ± 0.20	4.27 ± 0.30	3.73 ± 0.23	4.19 ± 0.16
VTL0714V	1. Top	2.40	2.40	4.43 ± 0.74	4.43 ± 0.79	5.59 ± 0.63	5.92 ± 0.78
	2. Left	2.39	2.39	5.35 ± 1.56	4.44 ± 0.65	7.00 ± 1.39	5.61 ± 0.43
	3. Center	2.39	2.40	4.26 ± 1.11	4.60 ± 1.40	5.40 ± 0.98	5.93 ± 1.41
	4. Right	2.39	2.39	4.14 ± 0.60	4.07 ± 0.31	5.52 ± 0.90	5.80 ± 2.00
	5. Bottom	2.40	2.40	4.53 ± 0.70	4.68 ± 2.39	5.92 ± 1.52	6.06 ± 2.78

Table S2. Cost of COWFISH2, related to Figure 1

Component	Product #	Manufacturer	Amount	Price (US$)*
**Imaging unit**				
machine vision camera	1800 U-2050m	Allied Vision	1	561
machine vision telecentric lens	VS-TCT1-65	VS Technology	1	1,086
φ1" emission quad-band pass filter	89402m	Chroma	1	596
lens filter mount	-	custom made	1	9
φ1" mirror	BB1-E02	Thorlabs	1	76
mirror holder	MH25	Thorlabs	1	17
mirror holder mount	KMS/M	Thorlabs	1	36
45° fixed stand	AM45C/M	Thorlabs	1	21
Cage System plate	CP37/M	Thorlabs	2	42
Cage System rod	ER3	Thorlabs	2	13
post rod	TR20/M	Thorlabs	2	10
post rod spacer	TR3M	Thorlabs	1	18
dovetail rail	RLA150/M	Thorlabs	1	46
dovetail rail carrier	RC1	Thorlabs	2	54
dovetail rail carrier small	RC4	Thorlabs	1	27
**Subtotal**				**2,612**
**Illumination unit**				
LED PCB, 470 nm	M470D4	Thorlabs	1	64
LED PCB, 625 nm	M625D3	Thorlabs	1	72

φ1" excitation filter, 480/20 nm	ET480/20x	Chroma	1	339
φ1" excitation filter, 635/20 nm	ZET635/20x	Chroma	1	339
φ1/2" aspheric condenser lens	ACL12708U-A	Thorlabs	2	58
φ1/2" lens holder	SM1A6T	Thorlabs	2	43
SM1 lens tube	SM1L10E	Thorlabs	2	31
SM1 lens tube ring	SM1RR	Thorlabs	2	9
SM1 lens tube mount	SM1RC/M	Thorlabs	2	50
LED heatsink	-	custom made	2	36
55° fixed stand	-	3D printed	2	1
**Subtotal**				**1,042**
**Stage unit**				
single-axis manual stage	XEG25	MISUMI	1	74
double-axis manual stage	XYEG40	MISUMI	1	152
stage bracket	-	custom made	1	55
stepper motor	PKP213D05A	Oriental motor	3	120
stage-motor adapter	-	3D printed	3	1
**Subtotal**				**402**
**Control device**				
ultra-mobile PC	GPD Pocket3 Ultimate	GPD	1	1,006
USB-PD adapter	CIO-G65W2C-BK	CIO	1	34
USB hub	4-IN-1-C	STRENTER	1	6
compatible Arduino Nano V3.0	JP-EL-CB-005	ELEGOO	1	8
1 A constant current LED driver	RCD-24-1.00	RECOM	2	23
stepper motor driver A4988	Pololu-1182	Pololu	3	53
**Subtotal**				**1,130**
**Others**				
breadboard	-	custom made	1	18
stage fixture	-	custom made	1	34
sample holder	-	custom made	1	110
exterior box	-	3D printed	1	26
other various small parts	-	-	-	50
**Subtotal**				**238**
**Total**				**5,424**

*: Prices were calculated based on the exchange rate at the time of acquisition in 2023 (140 JPY/US$).
